# 0737. Statins protect the vasculature from excessive angpt-2 production in sepsis

**DOI:** 10.1186/2197-425X-2-S1-P59

**Published:** 2014-09-26

**Authors:** K Thamm, C Ghosh, JT Kielstein, WC Aird, A Santel, SM Parikh, S David

**Affiliations:** Hannover Medical School, Hannover, Germany; Harvard Medical School, Center for Vascular Biology Research, Boston, USA; Silence Therapeutics AG, Berlin, Germany

## Introduction

Sepsis is a syndrome of systemic inflammation arising from infection that constitutes a top-ten cause of adult mortality. The recent withdrawal of a specific sepsis therapeutic has diminished pharmaceutical enthusiasm for developing novel drugs in this domain. Angiopoietin-2 (Angpt-2) is an endothelial-derived protein that potentiates vascular inflammation and permeability and may be involved in sepsis pathogenesis.

## Objectives

We set out to screen well-established drugs for their Angpt-2 lowering potential to ameliorate sepsis morbidity and to analyse the underlying molecular mechanism.

## Methods

### In vitro

FDA-approved library screening in human umbilical vein endothelial cells (HUVECs) and confirmation via Angpt-2 ELISA and quantitative RT-PCR.

### In vivo

Murine experimental sepsis was induced both by endotoxin (LPS) and cecal ligation and puncture (CLP). Mice were either treated with an Angpt-2 specific siRNA, simvastatin, or both and survival as well as the direct effect on Angpt-2 production was assessed by RT-PCR. In men: We analyzed circulating Angpt-2 levels in a retrospective matched case-control study in individuals with septic shock.

## Results

We found that simvastatin reduced endothelial Angpt-2 release and transcription in a time- and dose dependent manner in HUVECs. This effect required Simvastatin's HMG-CoA reductase activity. Similarly, *in vivo* simvastatin reduced the transcription of Angpt-2 murine lungs. In septic mice, specific inhibition of Angpt-2 in the pulmonary endothelium *via* an RNAi approach improved survival by 50% (p=0.002). Simvastatin equally improved survival, but the combination of Angpt-2 siRNA and simvastatin showed no additive benefit indicating that simvastatin might act *via* Angpt-2 inhibition. To investigate a potential link between statins and Angpt-2 in humans, we performed a matched case-control study in critically ill subjects and found that prior statin use was associated with lower circulating Angpt-2.

## Conclusions

In an unbiased approach Simvastatin was found to inhibit Angpt-2 production in vitro. This observation could be confirmed in vivo in different murine models of the disease. Our data indicate that a potential beneficial effect of prior statin use in septic humans might be promoted via the Angpt-2/Tie2 axis. Therefore, “point of care” screening for circulating Angpt-2 in candidates for a clinical sepsis trial might help to identify those individuals that benefit from a statin treatment.

